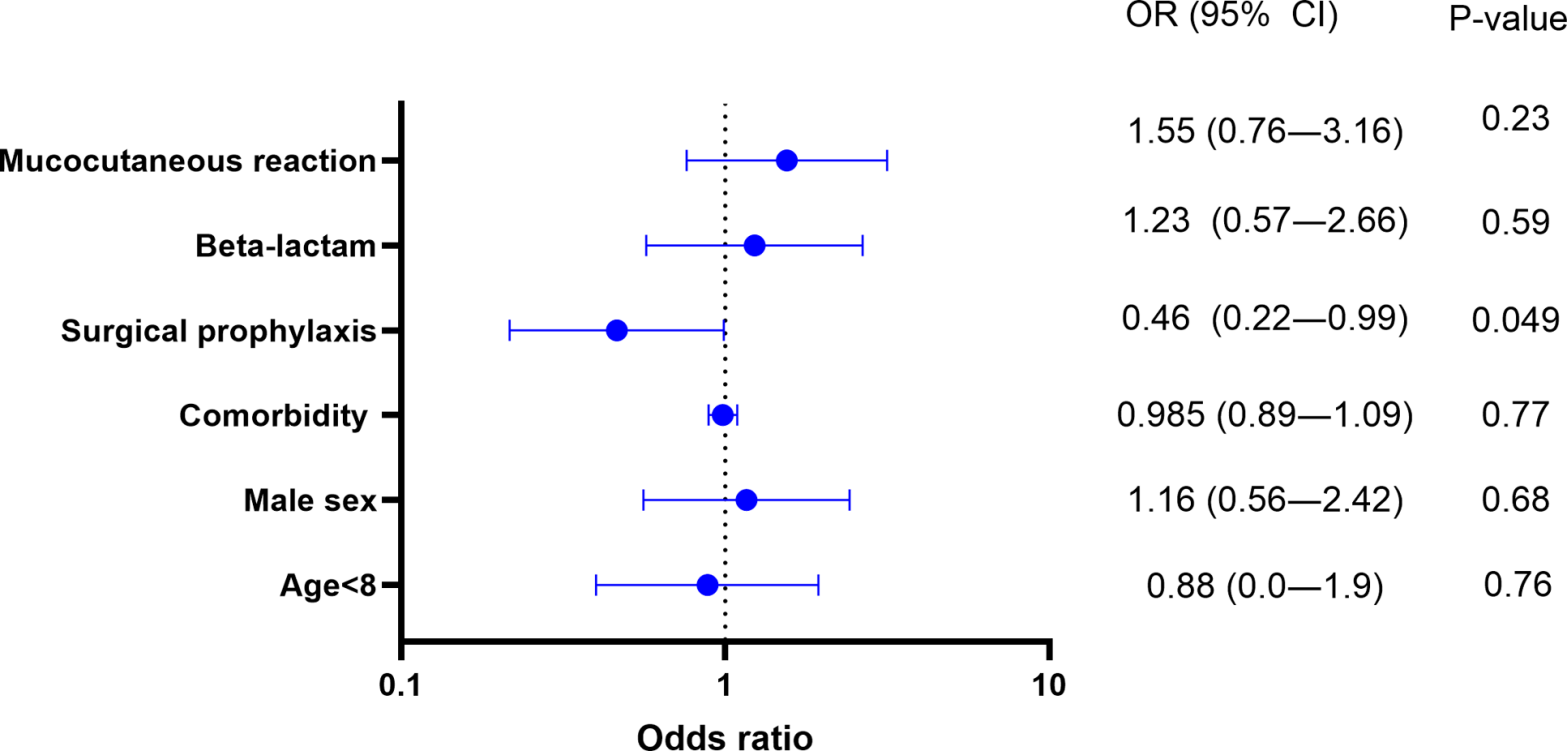# 60 Predictors of Hospital Penalties in the Hospital-Acquired Condition Reduction Program, Fiscal Year 2025

**DOI:** 10.1017/ash.2026.10491

**Published:** 2026-06-23

**Authors:** Ji Young Lee, Ju Yeon Park, Hye-Jung Choi, Hyeun Su Seo, Jee Yeon Baek, Jong Gyun Ahn, Ji-Man Kang

**Affiliations:** 1 Yonsei University College of Medicine; 2 Department of Pediatrics, Severance Children’s Hospital, Yonsei University College of Medicine

## Abstract

**Background:** Antibiotic allergies are frequently reported in children, yet most reactions are mild and should not preclude first-line therapy. Inaccurate allergy labels contribute to increased healthcare costs, Clostridioides difficile infection, and suboptimal or unnecessary antibiotic use. We aimed to characterize the incidence and features of inpatient antibiotic-associated allergic reactions, including serious events, and to determine the proportion of antibiotic regimens that were inappropriately modified in response to reported allergies. **Methods:** We conducted a retrospective study of patients ≤18 years admitted to Severance Children’s Hospital (Republic of Korea) during 2022–2024 who received oral or intravenous antibacterials and had an antibiotic-related adverse drug reaction (ADR) documented as “allergy” in the electronic health record. ADRs to antiviral, antifungal, or antiparasitic agents and reactions occurring in outpatient or emergency settings were excluded. Each discrete sign or symptom attributed to an antibiotic was counted as one allergic-reaction event. Management was considered appropriate if first-line therapy was continued for mild reactions, discontinued when unnecessary, aligned with international or national guidelines, or reflected pediatric infectious diseases expert opinion. **Results:** Among 31,164 admissions, 17,614 (56.0%) patients received antibiotics. A total of 215 antibiotic-related ADRs (0.92%) were documented, including 161 allergic-reaction events in 122 patients (0.7%) associated with 143 antibiotic prescriptions. Males accounted for 59.0% of cases; median age was 7.8 years (IQR, 2.8–12.4). The most common indications were surgical prophylaxis (34.3%), systemic febrile illness (14.0%), and pneumonia (11.2%). Frequent manifestations included nausea/vomiting (32.3%), urticaria (23.6%), and non-urticarial rash (18.1%). ?-lactams accounted for 67.3% of implicated agents; the most frequent drugs were ceftriaxone (26.8%), vancomycin (15.7%), and ampicillin/sulbactam (5.9%). Anaphylaxis occurred in 6 patients (5.0% of reactions; 0.002% of antibiotic prescriptions). Overall, 28.7% of allergy-labeled reactions were managed inappropriately, and 8.4% resulted in unnecessary escalation to broader-spectrum antibiotics (Table 1). Surgical prophylaxis was associated with reduced odds of inappropriate management compared with therapeutic use (OR, 0.46; 95% CI, 0.22–0.99; P **Conclusion:** True antibiotic allergy in hospitalized children is uncommon, yet nearly one third of reported allergies led to suboptimal or inappropriate antibiotic use. With the recent launch of the national antimicrobial stewardship pilot program in Korea (November 2024), we plan to develop an evidence-based clinical pathway for antibiotic allergy management and incorporate de-labeling strategies into the hospital electronic system to optimize antibiotic selection.

**Figure:**
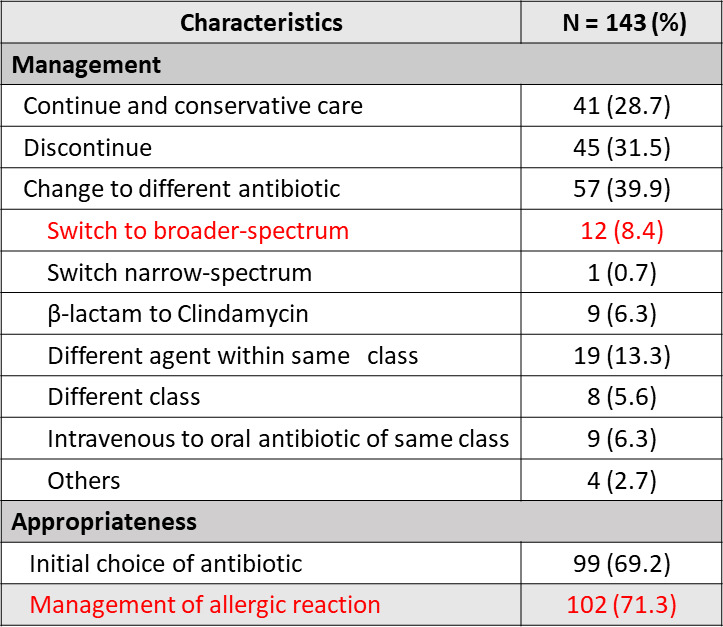

**Figure:**